# SILF Dataset: Fault Dataset for Solar Insecticidal Lamp Internet of Things Node

**DOI:** 10.3390/s25092808

**Published:** 2025-04-29

**Authors:** Xing Yang, Liyong Zhang, Lei Shu, Xiaoyuan Jing, Zhijun Zhang

**Affiliations:** 1 College of Intelligent Manufacturing, Anhui Science and Technology University, Chuzhou 233100, China; 2 NAU-Lincoln Joint Research Center of Intelligent Engineering, Nanjing Agricultural University, Nanjing 210031, China; 3 School of Engineering, University of Lincoln, Lincoln LN67TS, UK; 4 School of Computer, Guangdong University of Petrochemical Technology, Maoming 525000, China; 5 Guangdong Provincial Key Laboratory of Petrochemical Equipment Fault Diagnosis, Guangdong University of Petrochemical Technology, Maoming 525000, China; 6 School of Computer Science, Wuhan University, Wuhan 430072, China; 7 Provincial Key Laboratory for Green Agricultural Production and Intelligent Equipment, School of Environmental Science and Engineering, Guangdong University of Petrochemical Technology, Maoming 525000, China

**Keywords:** solar insecticidal lamp Internet of Things, fault detection and diagnosis, agricultural Internet of Things, outdoor scenarios, agricultural sensors, plant protection

## Abstract

Solar insecticidal lamps (SILs) are commonly used agricultural pest control devices that attract pests through a lure lamp and eliminate them using a high-voltage metal mesh. When integrated with Internet of Things (IoT) technology, SIL systems can collect various types of data, e.g., pest kill counts, meteorological conditions, soil moisture levels, and equipment status. However, the proper functioning of SIL-IoT is a prerequisite for enabling these capabilities. Therefore, this paper introduces the component composition and fault analysis of SIL-IoT. By examining long-term operational data from seven nodes deployed in real-world scenarios, different fault modes are identified. Six typical machine methods are adopted to verify the validity of the proposed dataset. The results indicate that machine learning algorithms can achieve high accuracy on the proposed dataset. Notably, voltage, current, and meteorological data play a crucial role in the fault diagnosis process for both SIL-IoT and other related agricultural IoT devices.

**Dataset:** Dataset has been deposited in IEEE DataPort as the following DOI: https://doi.org/10.21227/62z7-7s85, link: https://ieee-dataport.org/documents/silf-dataset-fault-dataset-solar-insecticidal-lamp-internet-things-node

**Dataset License:** CC-BY

## 1. Introduction

In recent years, agricultural pest outbreaks have been on the rise due to factors, e.g., rising temperatures and environmental pollution, leading to a decline in grain production and pose a significant threat to global food security. The Food and Agriculture Organization (FAO) estimates that pests destroy up to 40% of global crop production annually, exacerbating food insecurity for over 800 million people worldwide [1]. As a result, enhancing pest control in agriculture has become an urgent issue. One widely used agricultural pest control device is the frequency-vibration solar insecticidal lamp (SIL), which attracts pests with a lure lamp and eliminates them using a high-voltage metal mesh [2]. Statistical studies indicate that the deployment of SILs can reduce pesticide usage by 16% to 47%, decrease labor requirements by 14.1% to 32%, and contribute to environmental protection. Furthermore, the use of SILs can lower annual pest control costs by CNY 450 to 750 per hectare, thus enhancing food security and promoting ecological sustainability [3].

By integrating Internet of Things (IoT) technology, SIL evolves into the SIL-IoT system, which can acquire a variety of data, including the number of pests killed, agrometeorological conditions, soil moisture levels, and equipment status [4]. Each SIL-IoT device is equipped with wireless communication modules for data transmission, enabling real-time transfer of the collected information to a centralized backend [5]. This capability allows SIL-IoT to perform intelligent tasks, e.g., monitoring pest population levels. For example, by aggregating data from multiple devices, the system can pinpoint pest outbreak areas, helping plant protection personnel determine the optimal pesticide dosage and identify key treatment zones. Additionally, the data collected by SIL-IoT can be integrated into a comprehensive, multi-scale pest monitoring network that includes high-altitude monitoring lights, insect situation monitoring lights, insect radar, and other equipment. This network can analyze pest trends across different regions, ensuring timely interventions and preventing delays in pest control actions.

SIL-IoT performs critical tasks and has been widely studied in the following domain: the selection of lure lamps [6,7], node deployment strategy [8,9], energy management [10,11], monitoring the number of pests killed [12,13], and device maintenance [14,15]. However, factors such as aging, damage, and adverse weather conditions can cause component damage, software failure, and data anomalies, leading to the failure of the above functions [16]. This can result in misinterpretation of pest populations by both users and software platforms. Consequently, inaccurate data may lead to incorrect pesticide dosages, which can negatively affect crop quality and yield. To ensure the reliable operation of SIL-IoT, fault detection, and diagnosis (FDD) mechanisms are essential. FDD provides real-time insights into the device’s operational status, helping to maintain the accuracy of key data, e.g., the number of pests killed, local meteorological conditions, and remaining energy levels. These data are crucial for accurately identifying pest outbreak areas and estimating the appropriate pesticide usage.

Given the importance of maintaining reliable operation, research into the fault detection and diagnosis (FDD) of SIL-IoT is essential. FDD not only helps reduce the likelihood of component degradation due to faults but also ensures the integrity of acquired data, thereby improving the accuracy of subsequent data analysis and predictions for other tasks. Acquiring data under various fault conditions is crucial for performing the FDD of SIL-IoT. However, existing research on the FDD of SIL-IoT remains limited by the narrow fault scope (only focus on several failure modes), temporal sparsity (most collections lack continuous multi-season monitoring), and sensor isolation (few studies correlate electrical parameters with environmental conditions).

To address these gaps, we present the first SIL-IoT FDD dataset collected from seven SIL-IoT nodes deployed in outdoor environments. This dataset has advantages of comprehensive fault coverage (contains 15 single fault types and 32 coupling fault types), high-resolution temporal data (5-second interval recordings over 12 consecutive months), and multi-sensor synchronization (coordinated electrical, environmental, and pest count measurements), which will significantly advance research into SIL-IoT FDD and contribute to the optimization of its performance. In addition, this dataset may promote the following key advancements in agricultural IoT research: (a) cross-domain fault analysis linking environmental stressors to component degradation; (b) early fault detection algorithms using multivariate time-series patterns; and (c) predictive maintenance models based on cumulative operational stress indicators.

## 2. Dataset Description

Understanding the components of SIL-IoT and their operational characteristics is essential for grasping the fault mechanisms that may arise. Therefore, it is crucial to examine and discuss the potential fault conditions of SIL-IoT. This section begins by introducing the key components of SIL-IoT and outlining the functions they perform. It then presents five typical fault conditions, analyzing the specific data required to detect and diagnose each fault. Based on this analysis, the section further discusses the significance and diagnosability of these faults, emphasizing their impact on the system’s performance and reliability.

### 2.1. Component Introduction

**Figure 1:**
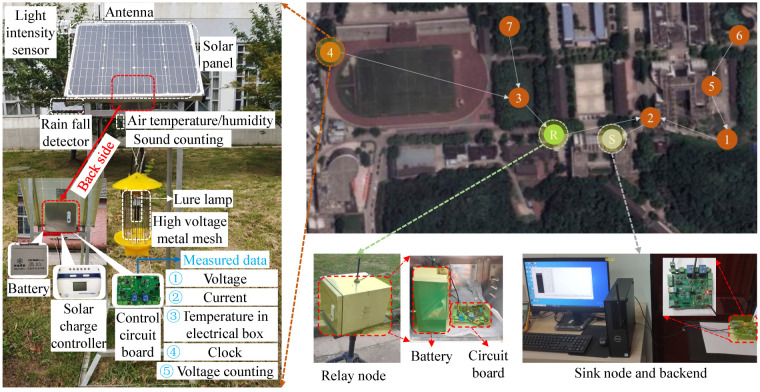
Component schematic diagram and real-world application of SIL-IoT, where the numbers 1–7 in the brown circle represent the ID of SIL-IoT.

As shown in Figure 1, SIL-IoT consists of several key components, including a solar panel (Trina Solar Co., Ltd., Changzhou, China), high-voltage metal mesh (Yangzhou Baodi Lighting Technology Co., Ltd., Yangzhou, China), lure lamp (Yangzhou Baodi Lighting Technology Co., Ltd., Yangzhou, China), battery (Trina Solar Co., Ltd., Changzhou, China), rain fall detector (self designed), control circuit board (self designed), pest storage box (Yangzhou Baodi Lighting Technology Co., Ltd., Yangzhou, China), stainless steel bracket (Yangzhou Baodi Lighting Technology Co., Ltd., Yangzhou, China), and IoT modules. The IoT modules are integral to the system’s functionality and include wireless communication module (ZigBee, BeiJing VeeYi Smart Tech Co. Ltd., Beijing, China), pest-counting modules (which can be divided into voltage-counting module and sound-counting module, Shenzhen Huameng Micro Technology Co., Ltd., Shenzhen, China), meteorological perception modules (various meteorological sensors, i.e., box temperature sensor, air temperature sensor, light-intensity sensor, and wind speed and direction sensor, Jinan Wufeng IoT Technology Co., Ltd., Jinan, China), component status monitoring modules (i.e., voltage and current sensors and clock chip, Shenzhen Zhiyi Technology Co., Ltd., Shenzhen, China), and computing chips (Arduino, Shenzhen Zhiyi Technology Co., Ltd., Shenzhen, China) [17].

**Figure 2:**
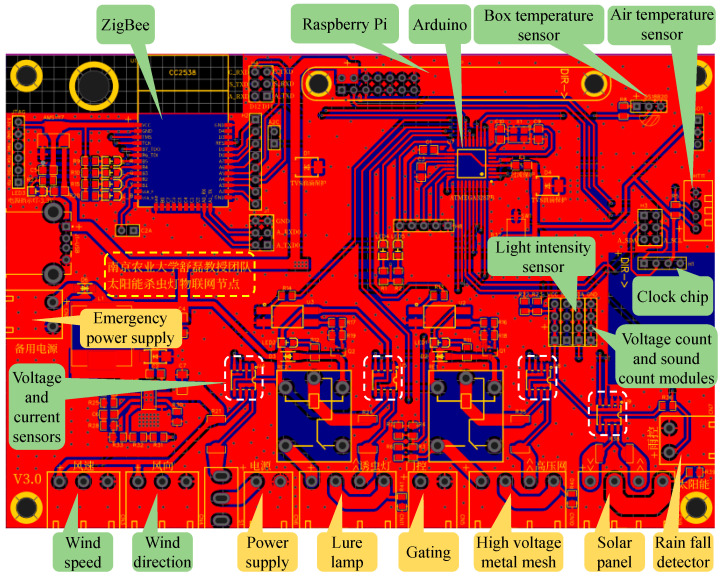
Connection of SIL module, IoT module, and PCB, where SIL module is illustrated in yellow box, and IoT module is shown in green box. The content in the yellow dashed box indicates that “This PCB of SIL-IoT node is designed by the Research Team of Professor Lei Shu from Nanjing Agricultural University, China”.

The connection between the above modules and the PCB is shown in Figure 2, the SIL module is illustrated in the yellow box, and the IoT module is shown in the green box. The function of the SIL system is based on the components described above. During the day, the solar panel harvests solar energy and charges the battery. At night, the lure lamp is activated to attract pests. When pests come into contact with the high-voltage metal mesh, high-voltage pulses are released to eliminate them. The rain fall detector monitors rainfall during the night, and, if rain is detected, the control circuit disables the lure lamp and high-voltage metal mesh to prevent energy waste. Finally, the pest corpses fall into the pest storage box, where they are either used as feed or analyzed by researchers to determine the pest population.

The IoT module performs several key functions. During the day, voltage and current sensors measure the energy harvested by the solar panels, allowing for an evaluation of the available power in the batteries for nighttime use. At night, the pest-counting module counts the number of pests killed. Multiple voltage and current sensors monitor the operational status of various components, ensuring proper functioning. Additionally, the meteorological perception modules continuously monitor environmental conditions around the SIL-IoT nodes, providing data that help quantify and predict pest occurrence patterns. The computing chip processes this information and issues control commands for activating the lure lamp and high-voltage metal mesh. It also handles other tasks, such as fault detection (FDD) and energy management, based on the data collected by the sensors. Finally, the wireless communication module transmits the acquired data and FDD results from the front-end system to the back end, enabling users to monitor the SIL-IoT’s status, track the number of pests eliminated, and access other relevant information.

### 2.2. Fault Situation Introduction

Based on research on equipment faults and maintenance records from multiple SIL-IoT, the typical fault situations can be divided as (1) control circuit faults caused by aging, corrosion, and other factors; (2) component damage, abnormal clock chip data, and rusting of rain fall detector leading to abnormal operation of lure lamp and high-voltage metal mesh; (3) the discharge performance of high-voltage metal mesh decreases due to the adhesion of pest corpses or conductive foreign objects; (4) damage and covering of solar panels, as well as aging of batteries leading to continuous insufficient residual energy; (5) abnormal data caused by sensor faults.

The first four fault scenarios can be remotely monitored using specific sensors. For abnormal data caused by sensor failures, FDD can be performed by analyzing the changing characteristics of the acquired data. The required sensor data are provided in Table 1. For component open circuit (OC) faults, the current change is significantly greater than the voltage change. Therefore, current measurements are taken for all components subject to FDD. The adhesion of pest corpses or foreign objects to the high-voltage metal mesh can lead to continuous discharge, resulting in high voltage fluctuations in the mesh and persistent voltage changes in the sound-counting module. By comparing the differences between the voltage and sound counts, foreign object adhesion faults in the high-voltage mesh can be detected. The temperature sensor in the electrical box is used to monitor the internal temperature, preventing potential battery damage due to excessive heat.

### 2.3. Fault Analysis

Designing targeted FDD methods based on the characteristics of different fault situations improves accuracy and efficiency [18]. Therefore, this section analyzes the importance and diagnosability of faults, as shown in Table 2.

#### 2.3.1. Fault Importance Analysis

Classifying different fault situations based on their impact and urgency is beneficial in prioritizing component maintenance. Faults are typically categorized into several levels of severity, i.e., slight, moderate, and serious faults [19].

- Slight faults have minimal impact on the system, allowing the equipment to operate normally. As a result, maintenance can be deferred or scheduled for a later time.
- Moderate faults still allow the equipment to function, but they may affect system performance. Timely repair and maintenance are necessary to prevent further degradation.
- Serious faults can lead to complete system failure or malfunction, requiring immediate emergency repairs to restore equipment operation.

For SIL-IoT, the most serious fault is its inability to function properly, e.g., being unable to turn on the lure lamp or eliminate pests. Fault situations that prevent SIL-IoT from operating include the following:

- An OC of the solar panel leads to no energy supply after battery power depletion.
- An OC of the lure lamp makes SIL-IoT unable to attract pests.
- An OC of high-voltage metal mesh makes SIL-IoT unable to eliminate pests after attracting them.
- A fault of the clock chip leads to abnormal local time data, which may cause the SIL-IoT to turn on the lure lamp and high-voltage metal mesh during the day and turn them off at night.

Based on the above, these fault situations are classified as serious faults. In addition, since such faults require prompt on-site maintenance, minimizing false positives is crucial to reducing the labor costs associated with equipment repairs.

Moderate faults have a certain impact on the performance of the SIL-IoT, but the equipment can still operate normally. Maintenance can be scheduled at a later time. This type of fault includes the following:

- A continuous discharge of high-voltage metal mesh causes a short-term decrease in the discharge voltage, influencing the accuracy of the insecticidal counting function. Moreover, after multiple occurrences of this fault situation, a large number of pest corpses may adhere to the high-voltage metal mesh, leading to a decrease in the attracting performance of the lure lamp.
- The dust covering of solar panels leads to a decrease in their power generation, affecting the battery life of SIL-IoT.
- An OC of light-intensity sensor, air temperature, and humidity sensor results in the inability to obtain corresponding meteorological observation data, which affects the execution of solar panel monitoring tasks, energy management tasks, and intelligent tasks that require relevant meteorological data.
- An OC of the temperature sensor in the electrical box makes it difficult to determine whether there is an abnormal temperature rise inside the electrical box. The above situation affects the execution of environmental monitoring tasks for the battery.

The impact of slight faults on SIL-IoT is reflected in abnormal data acquired by sensors. These faults can typically be resolved on the device side by resetting or recalibrating the device to restore the sensor to its normal working condition. This type of fault includes the following:

- An abnormal value of voltage and current sensors cause data to deviate from true values, which is not conducive to executing energy management and related component monitoring tasks.
- Abnormal data from the light-intensity sensor is not conducive to executing solar panel monitoring and related meteorological tasks.

Therefore, both fault situations are marked as slight faults. Maintenance personnel can correct the relevant abnormal data and remotely restart it for repair.

#### 2.3.2. Fault Diagnosability Analysis

The diagnosability of a fault represents the difficulty of accurately and effectively identifying the fault [20]. Considering constrained device computing resources and energy, this section primarily distinguishes the diagnosability of fault situations based on the required computing resources and data sources for FDD.

**Figure 3:**
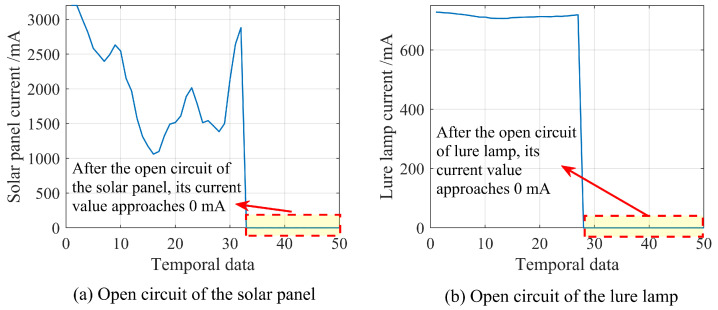
Examples of simple fault: (**a**) OC of the solar panel and (**b**) OC of the lure lamp, where the characteristics of both faults are significant.

Simple faults require minimal computing resources and rely solely on the device’s information to identify the fault causes. These faults typically show a significant difference in relevant characteristic values between the fault-free and fault states. Preliminary differentiation can be achieved through threshold settings or other methods [21]. As shown in Figure 3, the logic behind this type of fault is straightforward, and no further differentiation is needed based on the status of other devices or long-term temporal data. This type of fault includes the following:

- Solar panel OC, lure lamp OC, and high-voltage metal mesh OC can be diagnosed through voltage and current sensors. Among them, solar panel OC can only be diagnosed during daytime operation. Lure lamp OC and high-voltage metal mesh OC can only be diagnosed when the relevant components work at night.
- The continuous discharge of high-voltage metal mesh can be determined by the difference between the data from the voltage-counting module and the data from the sound-counting module.
- The OC of the light-intensity sensor, air temperature and humidity sensor, and temperature sensor in the electrical box are diagnosed by detecting the relevant measurement values reaching the threshold. Among them, the OC of the light-intensity sensor is only diagnosed during daytime operation.

The above faults can be diagnosed by studying FDD methods based on the device’s information. In addition, the above-mentioned faults are serious and moderate. Therefore, when designing FDD methods, it is necessary to minimize the occurrence of false positives.

There are two types of complex faults. The first type involves situations where the fault cause cannot be diagnosed solely based on the device’s information. These include issues such as the mismatch between the current value of the solar panel and the light-intensity value, the mismatch between the air temperature and the temperature in the electrical box, and clock chip failures. These faults are reflected as contradictory trends in two correlated temporal features.

**Figure 4:**
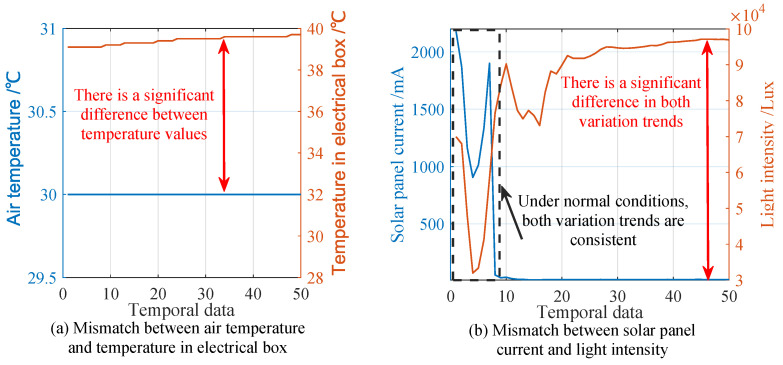
Examples of complex fault: (**a**) the mismatch between air temperature and temperature in electrical box, and (**b**) the mismatch between solar panel current and light intensity, where the root causes of both faults are difficult to identify through the device’s own short-term data.

For example, as shown in Figure 4a, the air temperature remains stable at 30 °C, yet the temperature in the electrical box is significantly higher. In this case, additional information from nearby devices is required to determine whether the abnormality lies with the air temperature or the electrical box’s temperature.

Figure 4b illustrates the mismatch in trends between the current value of the solar panel and the light-intensity value during a fault. These fault causes cannot be identified based on the device’s own data alone, meaning the faulty component cannot be directly pinpointed. Therefore, diagnosing such faults requires the integration of neighbor-node information and long-term temporal data.

Another type of complex fault arises from sensor failures, such as transient anomalies caused by data outliers, data gain, data stalling, data drift, or electromagnetic interference. The distinguishing features of these complex faults are often subtle, making it difficult to diagnose them solely by comparing data before and after the fault. As a result, setting a pre-defined threshold for FDD becomes challenging. Moreover, complex faults are influenced by factors such as the remaining energy of the equipment and individual differences between devices. These factors can lead to variations in the features of different equipment, further complicating FDD.

### 2.4. Data Format and Description

The dataset is obtained based on the setting in the next Section, which contained 502,916 rows of labeled samples with 41.8 MB and stored as “xlsx” file for easy reading. In addition, the dataset contains 23,966,722 rows with 2.80 GB and stored as “txt” file for saving storage spaces. The proposed dataset has been uploaded to IEEE DataPort [22]. The first row of each file denotes the corresponding feature name, which can be listed in Table 3.

## 3. Methods

Given that continuous FDD consumes energy and system resources, we configure the FDD method to operate as fixed-time triggers. The trigger times for the FDD method are determined by the clock chip’s time value, set to execute every three hours. Specifically, FDD occurs daily at 2:00, 8:00, 11:00, 14:00, 17:00, 20:00, and 23:00. Because the preset SIL turns off and the system restarts at 5:00, FDD during this time may be affected by voltage fluctuations and reset noise interference. In addition, FDD requires a certain amount of computing resources, which may lead to resource competition issues with the aforementioned processes. Therefore, FDD is not executed at 5:00. With this setup, each execution lasts for 5 min, during which the dataset retains only the first 5 min of data for each corresponding hour.

Since the data acquisition frequency of the SIL-IoT system is 5 s per reading, each measurement period captures 60 data points within those 5 min, including values for battery current, lure lamp current, high-voltage metal mesh current, and battery voltage. If an open-circuit (OC) fault occurs in any relevant component, the associated mismatch fault will not be labeled. For example, if the light-intensity sensor experiences an OC fault, the mismatch fault between the light-intensity value and the solar panel current value will not be labeled, reducing the likelihood of misdiagnosis.

Since the method does not rely on global information, the density of the SIL-IoT network remains unaffected as the number of nodes increases. Therefore, the seven existing nodes are sufficient to meet the application verification requirements. The start and end times across the seven nodes are provided in Table 4, where all samples are pre-processed and stored as “txt” format file. Nodes N4 and N7 stopped earlier than the others due to the conditions of the test site.

Due to the low frequency of pest infestations in winter and the adverse effects of low temperatures on SIL-IoT components, the nodes are recycled during winter and redeployed in the following summer. Accordingly, nodes N2, N3, and N6 were reclaimed on 9 November and redeployed to their original locations on 24 July. Nodes N1 and N5 simulate a scenario in which not all SIL-IoT nodes are recycled in winter, reflecting actual user usage patterns.

**Figure 5:**
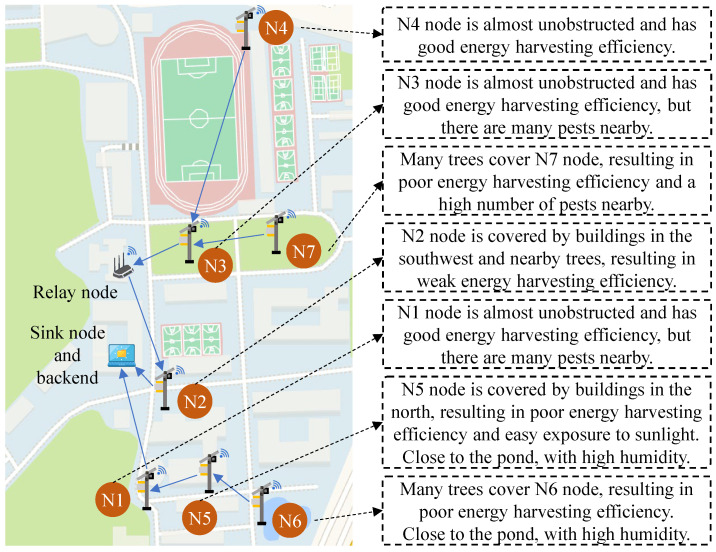
Deployment location and characteristics of seven SIL-IoT nodes, where all nodes are deployed at Nanjing Agricultural University, Nanjing, Jiangsu province, China, with the following longitude and latitude coordinates: N1 (118.706405, 32.137595), N2 (118.706635, 32.138447), N3 (118.707012, 32.139777), N4 (118.707079, 32.141585), N5 (118.707156, 32.137778), N6 (118.707654, 32.13761), and N7 (118.707861, 32.139731).

Figure 5 illustrates the locations and characteristics of the seven SIL-IoT nodes. In terms of lighting conditions, the nodes N1, N3, and N4 are less obstructed, allowing for sufficient energy collection. As a result, the probability of data anomalies caused by the clock chip failing to receive power from the battery is low.

In contrast, nodes N2, N5, N6, and N7 are frequently obstructed by buildings or trees, which can affect the performance of the light-intensity sensor and the solar panel. This may lead to discrepancies between the light-intensity readings and the current of the solar panel. Furthermore, due to the sun’s east-to-west path, the solar panels of N2, located on the northwest side of the building, are more likely to be shaded during the midday and afternoon hours. Similarly, the solar panels of N1, placed on the western side, are more prone to shading during noon. For nodes N5 and N4, which are deployed on the southern side of the building, shading is more likely in the morning. The occurrence of shading for nodes N3, N6, and N7, which are located around trees, is more random.

Regarding pest populations, nodes N1, N3, and N7 are deployed in grasslands with abundant vegetation, increasing the likelihood of a higher number of pests in these areas. Similarly, nodes N5 and N6, located near a pond, also face a higher probability of significant pest presence. As pests are eliminated by the high-voltage metal mesh, the likelihood of sustained discharge increases. Furthermore, the proximity of N5 and N6 to the pond raises humidity levels, which in turn increases the risk of component failure.

## 4. Technical Validation

### 4.1. Fault Result Analysis

This section categorizes various fault conditions in Table 2. Multiple faults may occur simultaneously, resulting in complex fault categories. The total number of faults across all seven nodes is summarized in Table 5 and Table 6. During maintenance, personnel may come into contact with wires, sensors, and other equipment, potentially leading to poor component connections. As a result, moderate and serious faults can occur intermittently. The last column of the table shows the frequency of different types of faults across all devices, while the last row represents the total number of faults recorded at each node.

F1 fault occurs mainly around 20:00 every day. The primary causes of F1 are low battery power, component damage, and aging. Due to the higher power consumption of the lure lamp compared to the high-voltage metal mesh, there are instances where the high-voltage metal mesh remains operational while the lure lamp either fails to turn on or turns on and off intermittently when the battery power is low. Although the lure lamp itself may not be damaged, such conditions are detrimental to its long-term performance. The N2 node, which is affected by building shadows in the afternoon, struggles to harvest sufficient energy, especially on rainy days, leading to low battery levels and increasing the likelihood of such issues.

The main reasons for F2 fault are decreased conductivity and delayed execution of control commands. Continuous adhesion of conductive objects, e.g., pest corpses, to the high-voltage metal mesh can reduce its conductivity. In extreme cases, a film layer may form on the mesh, preventing pests from discharging when they come into contact with it. In such situations, the current values at both ends of the high-voltage metal mesh approach zero, indicating an OC state. This is the primary cause of the OC fault in the high-voltage metal mesh reported by the N6 node. Additionally, to optimize energy use, all devices are programmed to activate the lure lamp via control commands at 19:00 every day, followed by activating the high voltage metal mesh within 10 s. However, if the clock chip data are shifted backward by one hour, the device will first turn on the lure lamp at 20:00. If the high-voltage metal mesh is activated more than 15 s after the lure lamp, the fault self-diagnosis system will identify this delay as a high-voltage metal mesh OC fault. This delay, observed around 20:00, is the main cause of the reported OC fault.

The fault that occurs most frequently in Table 5 is the clock chip fault. Clock chip faults are primarily caused by unexpected restarts due to battery depletion. For example, after the N1 node’s battery was depleted on 19 January 2022, the clock chip unexpectedly restarted, leading to a fault. Because the issue was not addressed in time, the fault persisted until 10 March 2022. During this period, the device experienced a situation in which the lure lamp and the high-voltage metal mesh were turned on during the day, but these components failed to activate on time at night. Similarly, the N2 and N3 nodes also encountered abnormal data due to unexpected restarts of their clock chips caused by battery depletion.

The F5 fault is caused by the device being in low battery levels for a long time or by the Raspberry Pi getting stuck, which prevents the device from executing tasks as scheduled. Due to insufficient lighting conditions, N2 and N6 nodess often shut down and restart due to a low battery. The above situation is the main reason for the F5 fault of N2 and N6 nodes.

The primary causes of F6 fault are loose contact, circuit aging due to improper maintenance, and incorrect wiring during installation, which can prevent the circuit from conducting properly. Furthermore, since temperature sensors are placed outside the electrical box, prolonged outdoor exposure increases the likelihood of damage. Nodes N1 and N5, having been in operation for a longer period, have a higher probability of experiencing OC faults in their air temperature sensors.

The main cause of F8 fault is continuous heating due to direct sunlight exposure on summer afternoons or a malfunction between the temperature sensor and the air temperature sensor. This type of fault occurs primarily in the afternoon. Among these, Nodes N3, N5, and N6 have a higher likelihood of experiencing such faults as they are continuously exposed to sunlight in the afternoon.

Nodes N1, N3, N5, and N6 have more frequent F11 faults due to being obstructed by buildings or trees. Abnormal current values in solar panels occur primarily from August to October, a period when lighting conditions are optimal, but the presence of obstructions causes significant variations in the current values.

The F9 fault occurs when pests or conductive foreign objects adhere to the mesh. This fault is critical for accurately tracking the number of pests eliminated and assessing the cleanliness of the mesh. Nodes N1 and N5 experience a higher frequency of this fault due to their significantly longer operating times compared to other nodes. The high frequency of this fault at node N6 can be attributed to its proximity to a pond, which increases the likelihood of a large number of pests and leads to more severe rusting of the metal mesh. This combination of factors makes it easier for pests to adhere to the high-voltage metal mesh.

**Figure 6:**
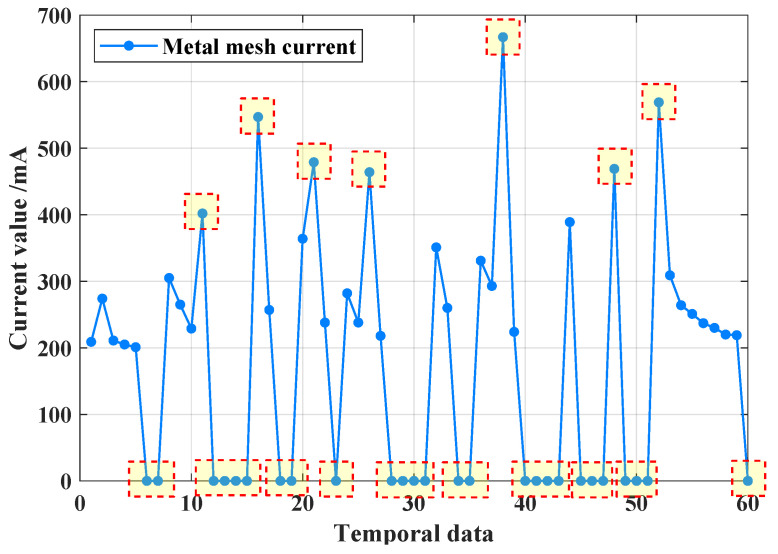
Multiple abnormal values when N1 node occurs a high-voltage metal mesh current outlier.

F12–F15 faults are primarily caused by frequent switching over a short period. The root cause of this frequent switching is poor contact at the end of the high-voltage metal mesh. When the mesh is briefly disconnected, accumulated energy is not properly released. Upon reconnection, this stored energy bursts out, leading to an abnormal spike in the current at both ends, which exceeds the normal operating current range, as shown in Figure 6.

**Figure 7:**
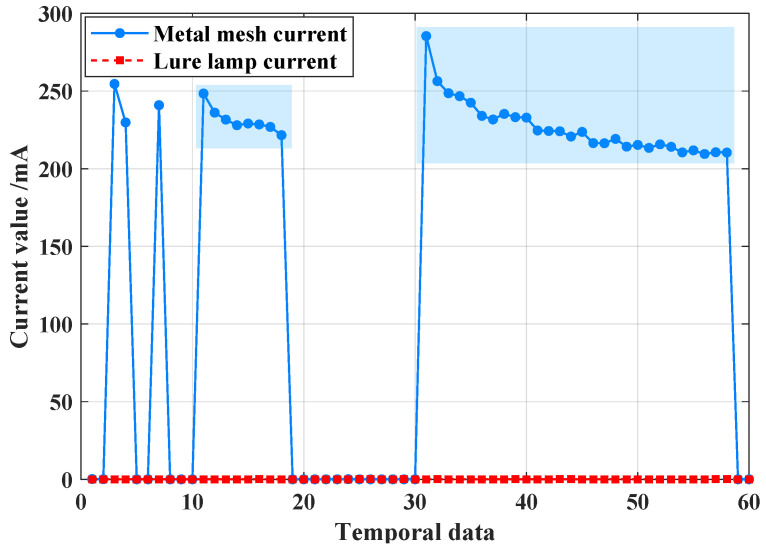
When N1 node occurs a high-voltage metal mesh current value abnormal, metal mesh current data changing between 0 and 200 mA.

The current gain in the high-voltage metal mesh follows a similar pattern to the outliers, except that the fluctuations in current do not exceed the normal operating range. As shown in Figure 7, the abnormal value of the current data from the high-voltage metal mesh is caused by issues such as sensor misalignment, resulting in a gradual increase in numerical deviation.

### 4.2. Validity Verification

**Figure 8:**
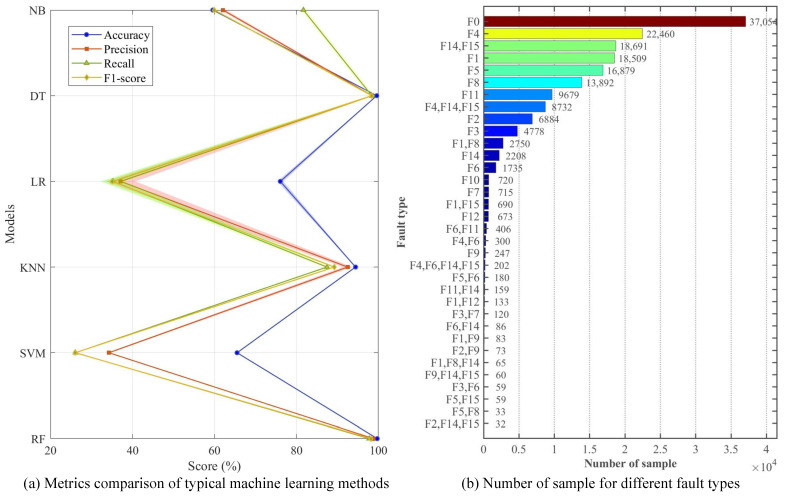
(**a**) Metrics comparison of typical machine learning methods and (**b**) number of sample for different fault types based on the dataset presented in this paper.

Six classic machine learning methods are adopted with a 5-fold cross-validation to validate the usability of the proposed dataset, including Random Forest (RF), Support Vector Machine (SVM), k-Nearest Neighbor (KNN), Logistic Regression (LR), Decision Tree (DT), and Naive Bayes (NB). All methods use default parameter settings. Due to the large number of fault-free (F0) samples, only 10% of F0 samples are used for training. In addition, the statistical significance of small sample categories is insufficient, which may not effectively support the model in learning discriminative feature patterns. Therefore, a small number of composite faults with a sample size of less than 20 are not included in the experiment. On this basis, a total number of 169,346 samples is used for training and testing. The sample ratio for each training and testing session is four to one. The average results are shown in Figure 8.

It should be emphasized that the purpose of this paper is to propose a suitable FDD dataset for SIL-IoT scenarios. Therefore, using machine learning methods for comparison is only for verifying the usability of the dataset, which is not the innovation point of this paper. The corresponding code has been uploaded to Github [23]. As shown in Figure 8a, the classification performance of the dataset in different models can be clearly evaluated, illustrating that the dataset has high accuracy on certain models.

From the experimental results, the RF performs the best among all models, achieving an average accuracy of 99.73%, indicating that this dataset can provide good support in handling complex classification tasks. In addition, the DT and KNN models demonstrate robust performance, achieving an average accuracy of 99.64% and 94.45%, respectively. This indicates that certain classic machine learning methods can fully capture the inherent patterns of data and effectively distinguish different categories for such datasets.

As shown in Figure 8b, although this paper has performed a simple sample balance, there is still a significant imbalance in the samples used for training and testing. Among them, the category with the largest sample size is F0, with a sample size of 37,054. The category with the largest sample size is compound fault “F2,F14,F15”, with a sample size of 32. Therefore, SVM and LR may lean towards majority classes, while RF naturally alleviates this problem through bootstrap sampling and Gini splitting criteria. In addition, SVM (especially the RBF kernel) is highly sensitive to feature scale. If not standardized, large-scale features will dominate kernel function calculations, while tree models are insensitive to scale. Considering that the proposed data may have non-linear relationships, linear models (e.g., LR) often perform poorly when dealing with non-linear problems. Through recursive splitting, local similarity measurement, and conditional probability decomposition, tree models (e.g., RF and DT) KNN and NB can better capture non-linear relationships, respectively. Based on the above reasons, the SVM and LR methods have lower classification accuracy in the proposed dataset.

**Figure 9:**
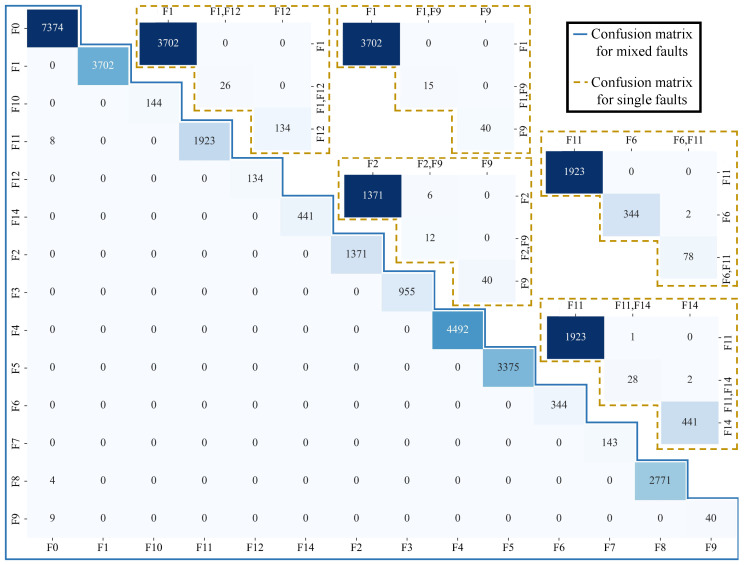
Confusion matrix of RF based on the dataset presented in this paper.

As shown in Figure 9, the confusion matrix of RF also illustrates the categoricity of the proposed dataset. Rows represent actual categories, while lists represent predicted categories. For example, the first row and the first column represent the number of samples that are actually F1 and predicted to be F1. The values on the diagonal represent the number of correctly classified samples, while the values on the diagonal represent the number of misclassified samples. The RF model can effectively identify the actual fault types of most samples. This indicates that the model has a certain practical value in FD. However, for some types of faults, e.g., F11 in a single fault, there may be certain errors in prediction, which may affect the reliability of the RF model in practical applications. In addition, for some combinations or single fault types, although there are many correct predictions, there are still a small number of samples that are misclassified. This requires further optimization of model parameters or improvement of model structure to enhance overall classification performance.

Despite most models achieving good classification performance, SVM and NB perform relatively weakly in accuracy and other performance metrics, i.e., precision and recall, with only 65.53% and 56.08%, respectively. This indicates that certain models may be confronted with problems of over simplification or inability to effectively capture complex patterns under the feature distribution of the current dataset. Therefore, future research on FDD models can further improve their classification accuracy in this dataset by optimizing algorithms and tuning the model hyperparameters. Future research can explore optimizing model structures, selecting more suitable features, and introducing new learning strategies to promote the application and development of FDD models of SIL-IoT.

### 4.3. Summary

Machine learning methods accurately identify faults on seven nodes, demonstrating its practical applicability to SIL-IoT. There is a notable correlation between the frequency of different faults and the characteristics of the nodes’ deployment locations. This suggests that diagnostic parameters for specific faults can be optimized based on deployment characteristics, allowing for more accurate and sensitive FDD, particularly for those faults that occur more frequently. In the actual maintenance process, eight faults required component replacement or circuit reconnection. The remaining serious and general faults were intermittent and were mainly attributed to poor circuit contact or similar problems. These faults can be automatically cleared shortly after the occurrence and addressed during routine inspections.

## 5. User Notes

As shown in Table A1, this dataset is valuable for the FDD of agricultural IoT devices. Voltage and current data are crucial for identifying abnormal conditions during equipment operation, while meteorological data help assess the influence of environmental factors on equipment faults. Pest data, particularly related to migratory pests, demonstrate its potential value in agricultural IoT applications. Beyond FDD, these datasets can also support tasks such as meteorological forecasting and pest tracking, further enhancing the accuracy and efficiency of agricultural production. Future research could focus on optimizing fault warning systems and integrating environmental and agricultural data to enable comprehensive management of agricultural IoT systems.

## 6. Conclusions and Future Work

SILs offer advantages such as their low cost, pollution-free operation, and self-sufficient energy. With IoT capabilities, SIL-IoT can acquire data on the number of killed pest, component status, and meteorological conditions. This study provides an in-depth analysis of the component composition and fault conditions of SIL-IoT, focusing on the fault modes and performance of seven nodes deployed over a long period. The results indicate that serious faults occur infrequently in SIL-IoT systems, while moderate and slight faults are more common. In the future, we will add sound and image capture devices on the basis of existing equipment to acquire diverse information. The fusion of numerical, sound, and image data contributes to detecting faults in SIL-IoT from different perspectives, thereby improving FD accuracy.

## Figures and Tables

**Table 1:** The sensor data required for above fault situations.

Fault Situations	Required Data	Corresponding Components
Control circuit faults	Voltage and current of different components	Lure lamp, solar panel, battery, and high-voltage metal mesh
Component damage	Clock chip time and rain fall detector voltage	Clock chip, light-intensity sensor, and rain fall detector
Discharge performance decrease	Voltage and sound count values	Voltage count and sound count module
Solar panel faults	Light-intensity value	Light-intensity sensor
Sensor faults	Long historical data	Sensors

**Table 2:** Fault name, identifier, importance, and diagnosability.

Fault Name	Identifier	Fault Importance	Fault Diagnosability
Fault-free	F0	/	/
Lure lamp OC	F1	Serious	Simple
Metal mesh OC	F2	Serious	Simple
Solar panel OC	F3	Serious	Simple
Clock chip fault	F4	Serious	Complex
system fault	F5	Serious	Complex
Air temperature sensor OC	F6	Moderate	Simple
Temperature sensor in electrical box OC	F7	Moderate	Simple
Mismatch between temperature value in electrical box and air temperature value	F8	Moderate	Complex
Metal mesh continuous discharge	F9	Moderate	Complex
Light-intensity sensor OC	F10	Moderate	Simple
Mismatch between light-intensity value and solar panel current value	F11	Moderate	Complex
Metal mesh current value abnormal	F12	Slight	Complex
Metal mesh voltage value abnormal	F13	Slight	Complex
Lure lamp current value abnormal	F14	Slight	Complex
Lure lamp voltage value abnormal	F15	Slight	Complex

**Table 3:** Dataset description.

Feature name	Data type	Example	Description
Time	datetime	16 September 2021 02:00:03	Time record for data acquisition
Node_ID	int	4	Used to identify different SIL-IoT nodes
Air_Temperature	int	24	Air temperature value acquired by DHT11
Related_Humidity	int	72	Air humidity value acquired by DHT11
Temperature_in _Electronic_Box	float	29.3	Temperature value in electronic box acquired by DS18B20
Battery_Voltage	float	12.84	Voltage value of battery acquired by 219A
Battery_Current	float	178.7	Current value of battery acquired by 219A
Metal_Mesh_Voltage	float	12.83	Voltage value of metal mesh acquired by 219A
Metal_Mesh_Current	float	103.6	Current value of metal mesh acquired by 219A
Lamp_Voltage	float	12.75	Voltage value of lure lamp acquired by 219A
Lamp_Current	float	718.1	Current value of lure lamp acquired by 219A
Solar_Voltage	float	12.3	Voltage value of solar panel acquired by 219A
Solar_Current	float	398.7	Current value of solar panel acquired by 219A
Light_Intensity	float	11,547.6	Light-intensity value acquired by MAX44009
Voltage_Count	int	1	Voltage fluctuation count when killing pests acquired by LM393
Sound_Count	int	8	Sound fluctuation count when killing pests acquired by FC-04
Rain_Voltage	int	1023	Rain fall detector voltage; if it does not exceed 900, it is recognized as rain
Fault	string	“F0”	Sample label (only existed in labeled data)

To display the format of all data under non-zero values, the examples shown in the table are not the same row of samples.

**Table 4:** Starting and ending times of FDD methods in seven nodes.

Node ID	Starting Time	Ending Time	Data Volume	Notes
N1	16 September 2021 00:00	16 September 2022 09:00	6,011,360	N2, N3, and N6 nodes were recalled on 9 November 2021 and redeployed on 24 July 2022.
N2	16 September 2021 00:00	5 December 2022 00:00	3,402,512	
N3	16 September 2021 00:00	27 November 2022 23:00	3,561,174	
N4	16 September 2021 00:00	18 September 2021 16:00	564,958	
N5	16 September 2021 00:00	19 November 2022 10:00	6,035,212	
N6	16 September 2021 00:00	15 December 2022 14:00	3,508,860	
N7	16 September 2021 00:00	4 October 2021 16:00	882,646	

**Table 5:** The number of single faults on seven SIL-IoT nodes.

Fault Type	N1	N2	N3	N4	N5	N6	N7	Total
F0	82,553	48,838	65,134	982	108,869	53,528	10,631	370,535
F1	118	1069	0	0	7358	9959	5	18,509
F2	1073	4499	80	0	37	1090	105	6884
F3	0	0	0	0	4788	0	0	4788
F4	12,798	9407	255	0	0	0	0	22,460
F5	2813	6187	2899	0	1385	3583	12	16,879
F6	449	0	35	0	831	420	0	1735
F7	0	0	655	0	60	0	0	715
F8	941	0	3577	36	6628	2602	108	13,892
F9	28	25	23	1	89	80	1	247
F10	0	0	0	0	0	0	720	720
F11	1724	675	836	60	5375	1003	6	9679
F12	79	75	145	1	261	101	11	673
F13	0	0	0	0	0	0	0	0
F14	1997	0	0	0	0	0	211	2208
F15	0	0	0	0	0	0	2	2
Total	104,573	70,775	73,639	1080	135,671	72,366	11,812	469,916

**Table 6:** The number of coupling faults on seven SIL-IoT nodes.

Fault Type	N1	N2	N3	N4	N5	N6	N7	Total
F1,F12	11	0	0	0	46	76	0	133
F1,F12,F15	0	0	0	0	0	0	4	4
F1,F15	0	0	0	0	0	0	690	690
F1,F8	345	0	446	0	1916	43	0	2750
F1,F8,F14	65	0	0	0	0	0	0	65
F1,F9	0	11	0	0	32	40	0	83
F1,F9,F12	0	0	0	0	0	3	0	3
F2,F8	0	0	0	0	0	8	0	8
F2,F9	71	1	0	0	1	0	0	73
F2,F14,F15	32	0	0	0	0	0	0	32
F3,F6	0	0	0	0	59	0	0	59
F3,F6,F11	0	0	0	0	20	0	0	20
F3,F7	0	0	0	0	120	0	0	120
F4,F6	300	0	0	0	0	0	0	300
F4,F6,F14,F15	202	0	0	0	0	0	0	202
F4,F12	6	0	0	0	0	0	0	6
F4,F6,F14,F15	10	0	0	0	0	0	0	10
F4,F14,F15	8732	0	0	0	0	0	0	8732
F5,F6	0	0	0	0	180	0	0	180
F5,F8	0	0	0	0	0	33	0	33
F5,F15	0	0	0	0	0	0	59	59
F6,F11	310	0	0	0	96	0	0	406
F6,F14	86	0	0	0	0	0	0	86
F6,F14,F15	13	0	0	0	0	0	0	13
F7,F12	0	0	5	0	0	0	0	5
F8,F9	0	0	1	0	0	1	0	2
F9,F12	1	2	1	0	1	8	0	13
F9,F14,F15	60	0	0	0	0	0	0	60
F11,F14	159	0	0	0	0	0	0	159
F12,F14	0	0	0	0	0	0	1	1
F12,F14,F15	2	0	0	0	0	0	0	2
F14,F15	18,663	0	0	0	0	0	28	18,691
Total	10,244	14	453	0	2471	212	753	14,147

## Data Availability

The original data presented in the study are openly available at [https://doi.org/10.21227/62z7-7s85].

## Appendix A

**Table A1:** Specifications table.

Subject	Agriculture Engineering
Specific subject area	Solar insecticidal lamps (SILs) are commonly used agricultural pest control devices that attract pests through a lure lamp and eliminate them using a high-voltage metal mesh. When integrated with Internet of Things (IoT) technology, SIL systems can acquire various types of data, e.g., pest kill counts, meteorological conditions, and equipment status. Voltage, current, and meteorological data play a crucial role in the FDD process for both SIL-IoT and other related agricultural IoT devices.
Type of data	Raw data, a total of 502,916 samples with 41.8 MB for labeled data in “xlsx” format and a total of 23,966,722 samples with 2.80 GB for unlabeled data in “txt” format
Data collection	All components are shown in Figure 1; the ZigBee technology was used for wireless transmitting data from nodes to back end. The time interval for data collection is set to 5 s.
Data source location	All nodes are deployed at Nanjing Agricultural University, Nanjing, Jiangsu province, China, with the following longitude and latitude coordinates: N1 (118.706405, 32.137595), N2 (118.706635,32.138447), N3 (118.707012,32.139777), N4 (118.707079,32.141585), N5 (118.707156,32.137778), N6 (118.707654,32.13761), and N7 (118.707861,32.139731).
Data accessibility	Repository name: IEEE DataPort
	DOI: https://doi.org/10.21227/62z7-7s85
	Direct URL to data: https://ieee-dataport.org/documents/silf-dataset-fault-dataset-solar-insecticidal-lamp-internet-things-node (accessed on 14 March 2025)
	Instructions for accessing these data: N/A
Related research article	N/A
Previously published dataset or data descriptor	N/A
